# miRNA Levels as a Biomarker for Anti-VEGF Response in Patients with Diabetic Macular Edema

**DOI:** 10.3390/jpm11121297

**Published:** 2021-12-04

**Authors:** Maartje J. C. Vader, Yasmin I. Habani, Reinier O. Schlingemann, Ingeborg Klaassen

**Affiliations:** 1 Ocular Angiogenesis Group, Department of Ophthalmology, Amsterdam Cardiovascular Sciences, Amsterdam Neuroscience, Amsterdam UMC, University of Amsterdam, Meibergdreef 9, 1105 AZ Amsterdam, The Netherlands; m.j.vader@amsterdamumc.nl (M.J.C.V.); y.i.habani@amsterdamumc.nl (Y.I.H.); r.o.schlingemann@amsterdamumc.nl (R.O.S.); 2 Department of Ophthalmology, University of Lausanne, Jules-Gonin Eye Hospital, Fondation Asile des Aveugles, Avenue de France 15, 1004 Lausanne, Switzerland

**Keywords:** diabetic macular edema, visual acuity, central area thickness, biomarker, microRNA, anti-VEGF

## Abstract

Background: The aim of this study was to investigate whether miRNA levels in the circulation could serve as a predictive biomarker for responsiveness to anti-vascular endothelial growth factor (VEGF) therapy in patients with diabetic macular edema. Methods: Whole blood samples were collected at baseline from 135 patients who were included in the BRDME study, a randomized controlled comparative trial of monthly bevacizumab or ranibizumab treatment for 6 months in patients with diabetic macular edema (Trialregister.nl, NTR3247). Best corrected visual acuity letter score (BCVA) and retinal central area thickness (CAT) were measured monthly during the 6-month follow-up. Levels of selected miRNAs were quantified. Results: Following linear regression analysis, the levels of four miRNAs were negatively associated with baseline CAT. Multivariable regression analysis confirmed this association for miR-181a. No associations with changes in CAT after 3 or 6 months of anti-VEGF treatment were found. In addition, no associations with miRNA levels with baseline BCVA or change in BCVA after 3 or 6 months of anti-VEGF treatment were found. Conclusions: Circulating miR-181a levels were negatively associated with CAT at baseline. However, no associations between miRNA levels and the response to anti-VEGF therapy were found.

## 1. Introduction

Patients with diabetic macular edema (DME) are commonly treated with intravitreal injections of anti-vascular endothelial growth factor (VEGF) agents. Unfortunately, a substantial number of patients exhibit a suboptimal response to anti-VEGF agents regarding the gain in visual acuity and might benefit from alternative therapeutics options, such as intravitreal corticosteroid injections or additional photocoagulation therapy [1,2,3]. To prevent long-term vision loss, it is important to identify this patient group at an early stage to ensure that they receive the proper treatment regimen. However, predictive biomarkers for anti-VEGF treatment response are lacking.

DME is the most important cause of visual impairment in patients with diabetic retinopathy. VEGF, the main known mediator of DME, is a protein secreted by retinal cells during hypoxia which is responsible for disruption of the blood–retinal barrier, resulting in fluid and protein leakage into the retina, causing DME, and eventually, visual impairment [4]. DME patients are therefore treated with intravitreal anti-VEGF agents, of which bevacizumab, ranibizumab and aflibercept are commonly used in clinical practice.

**Figure 1:**
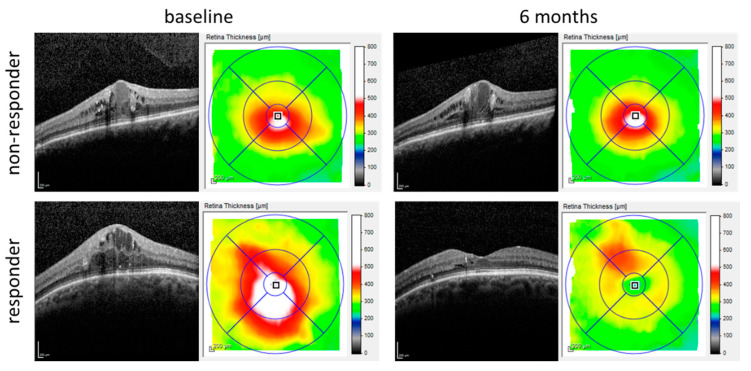
OCT images of DME patients obtained with Heidelberg Spectralis at baseline and after 6 months of anti-VEGF treatment. The central area thickness was unchanged after treatment in the non-responder patient, whereas the central area thickness was clearly decreased and macular edema was almost resolved in a responder patient.

However, up to 40% of DME patients treated with anti-VEGF agents exhibit a suboptimal response with minimal visual acuity gain or are non-responders. These patients would be better off switching to another anti-VEGF agent, or should initially be treated with alternative treatments [1,2,3]. Considering the varying response to anti-VEGF agents and the different treatment possibilities, treating clinicians need to know in advance whether a patient will be a responder or a non-responder (Figure 1). However, to date, no sufficient predictive biomarkers have been reported that are able to distinguish responders from non-responders.

Research on the role of microRNAs (miRNAs) in the pathogenesis of diabetes, diabetic retinopathy and diabetic macular edema is emerging. miRNAs are highly conserved, approximately 22 nucleotides long, noncoding RNAs that regulate gene expression. miRNAs are expressed in all human cells and are engaged in several physiological processes [5]. Distinct expression levels of specified miRNAs are known to be involved in the development of non-proliferative diabetic retinopathy and in the progression to proliferative diabetic retinopathy [5,6,7,8,9]. Only a few papers have reported a role for miRNAs in the development of DME [10,11,12]. However, it is unknown whether these miRNA expression profiles could also predict DME development or even predict responsiveness to anti-VEGF therapy.

The use of microRNA levels in circulation to predict therapy efficacy has previously been established in rheumatoid arthritis patients treated with anti-TNFα [13], and in the prediction of the response to radiotherapy in head and neck squamous cell carcinoma patients [14]. In the current study, we investigated whether miRNA levels in the circulation at baseline could serve as a biomarker for the responsiveness to anti-VEGF therapy in patients with diabetic macular edema. We selected seven miRNAs: miR-21, miR-181c and miR-1197, based on their reported roles as serum biomarkers for proliferative diabetic retinopathy (PDR) [8]; miR-181a and miR-222, for their roles in retinal neovascularization [15,16]; miR-133b, for its role in wound healing and the regulation of connective tissue growth factor (CTGF) [17]; and miR-7, for its role as a negative mediator of angiogenesis [18]. Using linear regression, we analyzed possible correlations between miRNA levels and the best corrected visual acuity letter score (BCVA) as well as central area thickness (CAT) at baseline and its change after 3- and 6-month follow-up.

## 2. Materials and Methods

### 2.1. Study Population

Patient material for miRNA analyses was obtained from participants of the BRDME study [19]. The BRDME study is a randomized, double-masked, multicenter clinical trial, conducted to generate conclusive evidence on the non-inferiority of bevacizumab to ranibizumab in the treatment of patients with DME (Trialregister.nl, NTR3247). The study was approved by the Institutional Review Board of the Amsterdam University Medical Centers, location AMC, and performed in accordance with the Declaration of Helsinki.

In the period from June 2012 to February 2018, 170 participants were enrolled in the BRDME study. Patients were eligible for participation if they presented vision loss due to DME and may benefit from treatment with anti-VEGF agents, were over 18 years of age, diagnosed with type 1 or type 2 diabetes mellitus with a glycosylated hemoglobin (HbA1c) of less than 12%, a CAT of >325 µm, and a BCVA between 24 and 79 letters. Over a period of 6 months, patients received monthly injections of either 1.25 mg bevacizumab (n = 86) or 0.5 mg ranibizumab (n = 84) for a follow-up time of 6 months.

**Figure 2:**
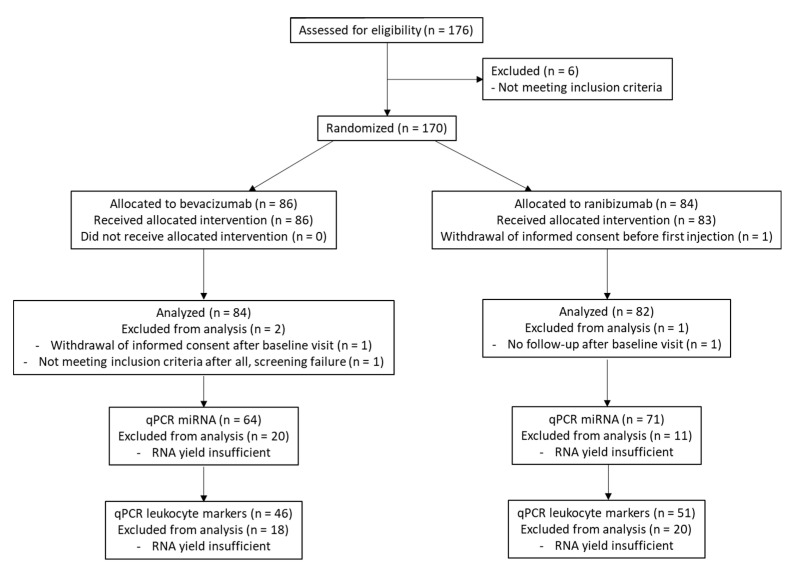
Flow chart summarizing the clinical design and data analysis.

The BCVA of the studied eye and CAT of the studied eye were measured monthly. BCVA was examined using the standardized Early Treatment Diabetic Retinopathy Study chart and registered as the number of letters read by the participant. CAT is defined as the 1 mm central retinal thickness area, as described in the ETDRS. Only at screening and after 6 months was a more comprehensive ophthalmic examination performed. Data on CAT were obtained from optical coherence tomography (OCT) scans, and different OCT devices were used depending on the available system at the participating center (Zeiss Cirrus, Heidelberg Spectralis or Topcon). CAT values obtained from Zeiss Cirrus and Topcon devices were converted to Heidelberg Spectralis outcomes, according to the conversion table derived by Giani et al. [20]. Blood samples for DNA and RNA extraction were collected during the screening visit, before administration of the first injection. The diagnosis of DME was confirmed by an independent reading center, the Belfast Reading Center, Belfast, United Kingdom, part of the Network of Ophthalmic Reading Centers of the United Kingdom. A summary of the clinical design and data analysis is given in Figure 2.

### 2.2. Outcome Measurements

The primary outcomes included the association between miRNA expression levels and the change in BCVA from baseline to 3 and 6 months, and the association between miRNA levels and change in CAT from baseline to 3 and 6 months. In addition, differences in miRNA expression levels between responder groups were examined. Participants were appointed to one of the following groups based on changes in CAT: rapid response, defined as a reduction of >50% in CAT after 3 months of treatment; slow response, reduction in CAT between 10% and 50% at 3 months, but a reduction of >50% at 6 months; and no response, a less than 25% reduction in CAT at both 3 and 6 months.

### 2.3. miRNA Profiling

Whole blood samples of 2.5 mL were collected from 135 participants, which were drawn into PAXgene RNA tubes; eventually, RNA was extracted using the PAXgene blood RNA kit (Qiagen, Hilden, Germany), following the manufacturer’s instructions. miRNA expression profiles were quantified using real-time quantitative reverse transcription polymerase chain reaction (qRT-PCR). A 1 μg aliquot of total RNA of each sample was reverse-transcribed into cDNA using the miScript II RT Kit (Qiagen). The samples were diluted 20 times with nuclease-free water. Real-time qPCR was performed using a CFX96 real-time PCR detection system (Bio-Rad Laboratories, Hercules, CA, USA). A master mix was prepared for each primer set, consisting of a 5 μL iQ SYBR green supermix (Bio-Rad) and 1 μL primer mix. Additionally, 4 μL of cDNA (diluted 1:80) in 6 μL master mix was amplified using the following PCR protocol: 95 °C for 10 min and 60 °C for 1 min, followed by 44 cycles of 95 °C for 10 s and 60 °C for 1 min, followed by 95 °C for 30 s and a melting program (60–95 °C). Calculating the relative gene expression, the equation: R = E^−Ct^ was used, where E is the mean efficiency of all samples as determined by LinReg [21], and Ct is the threshold for the gene that was determined during qPCR. The following miRNAs were analyzed in our study cohort: miR-7, miR-21, miR-133b, miR-181a, miR-181c, miR-222 and miR-1197. These were selected based on reported associations with angiogenesis, diabetes or diabetic retinopathy [5,6,8]. Primers were purchased from Exiqon. All reactions were performed in quadruplicates. Gene expression data were normalized using UnisP6.

### 2.4. mRNA Expression of Leukocyte Markers

The mRNA levels of the following leukocyte markers were analyzed: PTPRC/CD45 (pan-leukocyte marker), CD4 (CD4^+^ T-cells), CD8A (CD8^+^ T-cells), CD19 (B-cells) and CD14 (monocytes). A 1 μg aliquot of total RNA of each sample was treated with DNase-I (amplification grade; Invitrogen, Waltham, MA, USA) and reverse-transcribed into cDNA using the Maxima First Strand cDNA Synthesis Kit (Thermo Scientific, Waltham, MA, USA). Real-time quantitative PCR (RT-qPCR) was performed on 20× diluted cDNA samples using a CFX96 real-time PCR detection system (Bio-Rad Laboratories) and the specificity of primers was confirmed as described previously [22]. Primer details are available upon request. Gene expression data are expressed as absolute amounts. All reactions were performed in triplicates.

### 2.5. Statistical Analysis

Linear regression analysis was performed to evaluate the relationship between miRNA levels and baseline BCVA, changes in BCVA, baseline CAT and changes in CAT from baseline to 3 and 6 months. miRNA and mRNA data were log10-transformed to obtain a normal distribution. Multivariable regression analysis was used to confirm associations found by univariable regression analysis.

For responder and non-responder analyses, a threshold of 283 µm was subtracted from OCT values, which equaled the mean normal retinal thickness, as measured with Heidelberg Spectralis, because all OCT measurements were converted to Heidelberg Spectralis values [20]. Relative changes in CAT were used for the classification of rapid responders, slow responders and non-responders, as described in Section 2.2. For responder analysis, miRNA levels were compared using the Kruskal–Wallis test with multiple comparisons.

Significance levels of <0.05 were considered as statistically significant in all analyses mentioned above.

## 3. Results

### 3.1. Patient Characteristics

The mean age of the patients in the study was 63.7 ± 11.4 years (±standard deviation); 85 were male and 51 were female. The majority of the participants (*n* = 119) were diagnosed with type 2 diabetes mellitus, whereas 16 participants were diagnosed with type 1 diabetes mellitus. The mean duration since the diagnosis of diabetes mellitus was 16.0 ± 11.1 years. The mean baseline BCVA score was 68.6 ± 9.8 letters, and participants had a mean CAT of 462.5 ± 101.3 µm. Hemoglobin A1c (HbA1c) levels of only 60 patients were documented, with a mean HbA1c of 7.78%.

### 3.2. miRNA Levels and Visual Acuity Outcomes

Following linear regression analysis, levels of miRNAs in the circulation at baseline were not associated with BCVA at baseline (Table 1). In addition, no association with miRNA levels or change in BCVA at 3 and 6 months was found.

### 3.3. miRNA Levels and Central Area Thickness

**Figure 3:**
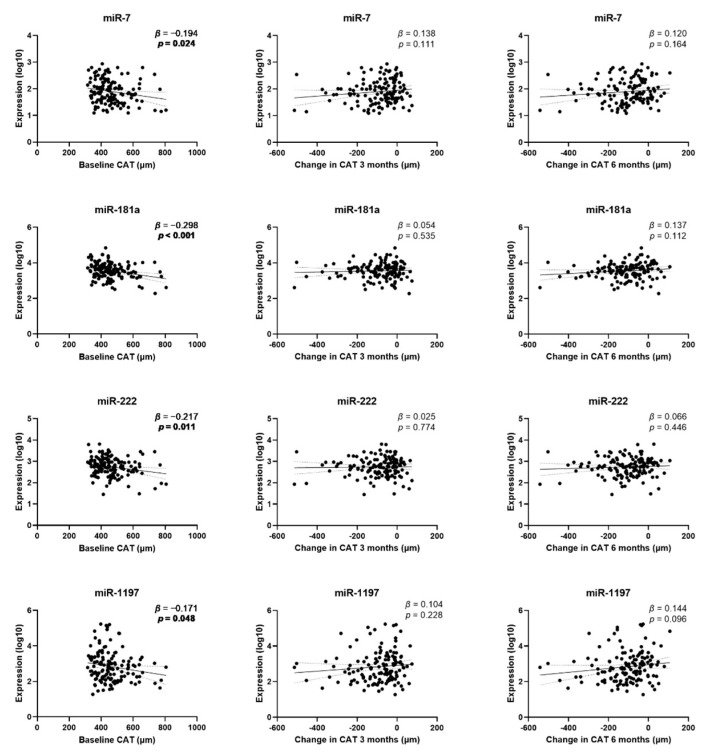
Association between circulating miRNAs and baseline CAT or change in CAT at 3 and 6 months. The scatter plots show the simple linear regression line with 95% confidence bands. Beta (*β*) coefficients and *p*-value (*p*) are given. *p*-values < 0.05 are indicated in bold.

Following linear regression analysis, CAT at baseline was negatively associated with miRNA levels in circulation at baseline for miR-7, miR-181a, miR-222 and miR-1197 (Table 2, Figure 3). Multivariable linear regression analyses confirmed a significant association of miR-181a with baseline CAT. No associations of any miRNA levels and change in CAT at 3 or 6 months could be demonstrated (Table 2, Figure 3).

### 3.4. Responder Analyses

**Figure 4:**
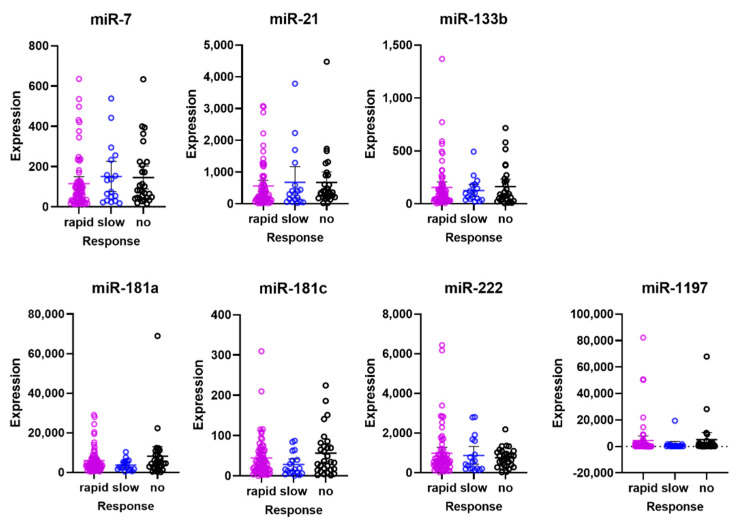
Expression of circulating miRNAs at baseline in different responder groups. Rapid responders exhibited a reduction of >50% in CAT after 3 months of treatment, slow responders exhibited a reduction in CAT between 10% and 50% at 3 months but a reduction of >50% at 6 months, and patients with no response exhibited a less than 25% reduction in CAT at both 3 and 6 months.

Following the criteria for responsiveness as described in the Methods section, 66 patients were defined as rapid responders, 18 patients as slow responders, and 29 patients as non-responders. Using the Kruskal–Wallis test, no significant differences were found in miRNA levels between these responder groups (Figure 4).

### 3.5. miRNA Levels and Leukocyte Markers

The majority of mRNA isolated from whole blood comes from leukocytes, and these cells play an important role in the pathogenesis of DME; therefore, we checked whether the expression of leukocyte markers is related to miRNA expression and VA and CAT outcomes. The following leukocyte markers were included in the analyses: CD4, CD8a, CD14, CD19 and CD45.

**Figure 5:**
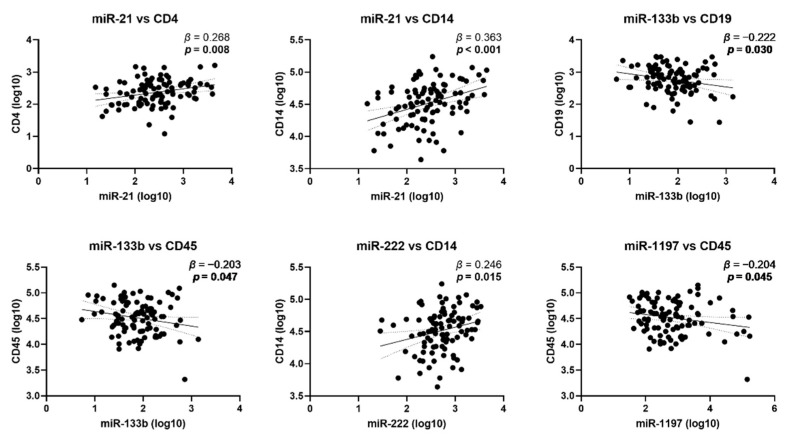
Association between circulating miRNAs and leukocyte markers at baseline. The scatter plots show the simple linear regression line with 95% confidence bands. Beta (*β*) coefficients and *p*-values (*p*) are given. *p*-values < 0.05 are indicated in bold.

Regression analysis demonstrated a number of associations between miRNA levels and leukocyte markers (Figure 5). miR-21 was positively associated with the marker of CD4^+^ T-cells CD4 and the monocyte marker CD14. miR-133b was negatively associated with the B-cell marker CD19 and the pan-leukocyte marker CD45. miR-222 was positively associated with CD14 and miR-1197 was negatively associated with CD45. Expression levels of leukocyte markers were not associated with BCVA or CAT at baseline or with CAT changes at 3 or 6 months.

## 4. Discussion

The use of microRNA levels as biomarkers for disease progression or in the prediction for therapy response is emerging [13,14]. In the present study, we compared the expression levels of selected miRNAs with BCVA and central area thickness (CAT) at baseline and after 3 and 6 months of anti-VEGF therapy. Four miRNAs, miR-7, miR-181a, miR-222 and miR-1197, exhibited a negative association with baseline CAT, as analyzed with univariable regression analysis. For miR-181a, this association could be confirmed with the use of multivariable regression analysis. No associations were found between miRNA levels and changes in CAT or BCVA after therapy, which means that these miRNAs are not suitable as predictors for therapy efficacy. We could also not demonstrate an association between miRNA levels and baseline BCVA. Therefore, expression in the circulation of the studied miRNAs does not seem to be related to the DME response to anti-VEGF.

The main positive finding of this study is that levels at baseline of miR-181a are negatively associated with baseline CAT, suggesting that miR-181a levels decrease when CAT increases. This finding seems in line with a recent study which reported that miR-181a is enriched in the normal mouse retina, but reduced in the retinas of mice in an oxygen-induced retinopathy (OIR) model [15]. The downregulation of miR-181a was accompanied by the upregulation of its target gene endothelial cell-specific molecule 1 (endocan or ESM-1), which has previously been identified to be highly enriched in retinal endothelial tip cells [23], and which has been shown to play an important role in the regulation of angiogenesis [24]. The intraocular injection of miR-181a resulted in the suppression of retinal neovascularization in the OIR model [15].

In addition, extracellular microvesicles (EVs) derived from apoptotic human T lymphocytes (LMPs) were found to have anti-angiogenic properties in the OIR model, and miR-181a was shown to be one of the most abundant miRNAs in these LMPs [25]. This confirms an anti-angiogenic role for miR-181a and suggests LMPs as a source of this miRNA, which may also explain our findings in the circulation.

To explore this further, we investigated possible associations between mRNA levels of leukocyte markers and miRNA levels. We were unable to find a relationship between miR-181a and the abundance of T-cells with markers for CD4 and CD8A, however, suggesting that leukocytes may not be the source of miR-181a, or that the used method is not appropriate. However, associations with leukocyte markers could be established for miR-21, miR-133b, miR-222 and miR-1197, suggesting that leukocytes may be a source for these miRNAs. Other sources of miRNAs may be diverse, including passive leakage from inflamed or injured cells or platelets, active secretion via cell-derived membrane vesicles such as microparticles, exosomes, shedding vesicles, and apoptotic bodies, and active secretion by a protein–miRNA with lipoproteins (e.g., high-density lipoprotein: HDL) and Argonaute protein (e.g., Ago2) [26].

We used whole serum samples; therefore, we could not distinguish between miRNAs from these different sources, although miRNAs derived from leukocytes were expected to be the most abundant in our samples. It remains to be determined which sources of circulating miRNAs can best be used as biomarkers for the diagnosis, prevention, and treatment of disease, but thus far, research is in favor of exosomal samples compared to whole serum samples [26]. Future experiments with exosomal-derived miRNAs from serum may discover better associations with therapy efficacy in DME than we found in the current study.

In addition to the lack of an association of miRNA levels with changes in CAT or BCVA after therapy, we could also find no differences between the different responder groups. In our study, we used selected miRNAs. Possibly, by using a discovery-based method and analyzing all possible miRNAs available, future studies will reveal other miRNAs that do associate with therapy efficacy.

Another limitation of this study is that we only had blood samples collected at the beginning of the study, before the start of anti-VEGF treatment. The expression of miRNAs can change rapidly in response to changes in the microenvironment; therefore, a single measurement is only a snapshot and may not truly reflect the dynamic regulation of miRNAs in relation to changes in BCVA and CAT after therapy. To better understand the role of these miRNAs in the response to anti-VEGF treatment, blood samples should also be examined during and after treatment in future studies.

## 5. Conclusions

Our findings demonstrated no role for the miRNAs studied in predicting response to anti-VEGF therapy. However, we did observe an association between miR-181a levels and CAT at baseline. Future research is needed to further investigate the predictive value of miRNAs as biomarkers of anti-VEGF treatment response, and to fully understand the role of miRNAs in the pathophysiology of DME.

## Figures and Tables

**Table 1:** Linear regression analysis for log10-transformed miRNA levels and baseline BCVA and changes in BCVA at 3 and 6 months.

	miRNA	miR7	miR21	miR133b	miR181a	miR181c	miR222	miR1197
Baseline BCVA	Beta	0.132	0.152	−0.047	0.102	0.080	0.154	0.078
	SE	1.868	1.486	1.782	1.904	1.345	1.911	0.990
	*p*-value	0.128	0.078	0.586	0.240	0.354	0.074	0.371
∆BCVA 3 months	Beta	−0.135	−0.088	0.068	0.020	0.046	−0.041	−0.040
	SE	1.299	1.042	1.238	1.331	0.938	1.344	0.690
	*p*-value	0.118	0.310	0.430	0.821	0.579	0.635	0.642
∆BCVA 6 months	Beta	−0.062	0.004	0.167	0.108	0.081	0.060	0.008
	SE	1.437	1.149	1.343	1.453	1.027	1.475	0.758
	*p*-value	0.473	0.962	0.053	0.214	0.353	0.490	0.922

Beta, beta coefficient; SE, standard error.

**Table 2:** Univariable and multivariable linear regression analysis for log10-transformed miRNA levels and baseline CAT and changes in CAT at 3 and 6 months.

		miRNA	miR7	miR21	miR133b	miR181a	miR181c	miR222	miR1197
Baseline CAT	Univariable	Beta	−0.194	−0.154	−0.151	−0.298	−0.157	−0.217	−0.171
		SE	19.132	15.373	18.244	18.906	13.792	19.538	10.119
		*p*-value	0.024	0.074	0.080	0.000	0.070	0.011	0.048
	Multivariable	Beta	−0.083	0.013	−0.002	−0.348	0.070	0.097	−0.106
		SE	30.418	27.553	22.193	33.824	18.543	43.481	10.780
		*p*-value	0.540	0.932	0.986	0.020	0.546	0.608	0.246
∆CAT 3 months	Univariable	Beta	0.138	0.058	0.001	0.054	−0.028	0.025	0.104
		SE	21.117	16.982	20.178	21.619	15.260	21.876	11.167
		*p*-value	0.111	0.506	0.993	0.535	0.747	0.774	0.228
	Multivariable	Beta	0.273	0.034	−0.074	0.168	−0.129	−0.259	0.113
		SE	34.108	30.896	24.886	37.928	20.793	48.757	12.088
		*p*-value	0.051	0.827	0.493	0.270	0.278	0.182	0.226
∆CAT 6 months	Univariable	Beta	0.120	0.053	0.010	0.137	0.018	0.066	0.144
		SE	21.871	17.553	20.850	22.160	15.773	22.563	11.482
		*p*-value	0.164	0.541	0.913	0.112	0.839	0.446	0.096
	Multivariable	Beta	0.191	−0.009	−0.091	0.286	−0.122	−0.218	0.142
		SE	35.111	31.804	25.617	39.043	21.404	50.190	12.444
		*p*-value	0.169	0.957	0.394	0.061	0.302	0.259	0.128

Beta, beta coefficient; SE, standard error.

## Data Availability

Raw data were generated at the Department of Biology, KU Leuven, and the Department of Ophthalmology, Amsterdam UMC. Derived data supporting the findings of this study are available from the corresponding author (I.K.) on request.

## References

[1] Chatziralli I. (2018). Editorial-Suboptimal response to intravitreal anti-VEGF treatment for patients with diabetic macular edema: Is there any point in switching treatment?. Eur. Rev. Med. Pharmacol. Sci..

[2] Chen Y.P., Wu A.L., Chuang C.C., Chen S.N. (2019). Factors influencing clinical outcomes in patients with diabetic macular edema treated with intravitreal ranibizumab: Comparison between responder and non-responder cases. Sci. Rep..

[3] Gonzalez V.H., Campbell J., Holekamp N.M., Kiss S., Loewenstein A., Augustin A.J., Ma J., Ho A.C., Patel V., Whitcup S.M. (2016). Early and Long-Term Responses to Anti-Vascular Endothelial Growth Factor Therapy in Diabetic Macular Edema: Analysis of Protocol I Data. Am. J. Ophthalmol..

[4] Witmer A. (2003). Vascular endothelial growth factors and angiogenesis in eye disease. Prog. Retin. Eye Res..

[5] Mastropasqua R., Toto L., Cipollone F., Santovito D., Carpineto P., Mastropasqua L. (2014). Role of microRNAs in the modulation of diabetic retinopathy. Prog. Retin. Eye Res..

[6] Gong Q., Su G. (2017). Roles of miRNAs and long noncoding RNAs in the progression of diabetic retinopathy. Biosci. Rep..

[7] Liu H.N., Li X., Wu N., Tong M.M., Chen S., Zhu S.S., Qian W., Chen X.L. (2018). Serum microRNA-221 as a biomarker for diabetic retinopathy in patients associated with type 2 diabetes. Int. J. Ophthalmol..

[8] Qing S., Yuan S., Yun C., Hui H., Mao P., Wen F., Ding Y., Liu Q. (2014). Serum miRNA biomarkers serve as a fingerprint for proliferative diabetic retinopathy. Cell Physiol. Biochem..

[9] Friedrich J., Steel D.H.W., Schlingemann R.O., Koss M.J., Hammes H.P., Krenning G., Klaassen I. (2020). microRNA Expression Profile in the Vitreous of Proliferative Diabetic Retinopathy Patients and Differences from Patients Treated with Anti-VEGF Therapy. Transl. Vis. Sci. Technol..

[10] Jiang L., Cao H., Deng T., Yang M., Meng T., Yang H., Luo X. (2021). Serum exosomal miR-377-3p inhibits retinal pigment epithelium proliferation and offers a biomarker for diabetic macular edema. J. Int. Med. Res..

[11] Grieco G.E., Sebastiani G., Eandi C.M., Neri G., Nigi L., Brusco N., D’Aurizio R., Posarelli M., Bacci T., De Benedetto E. (2020). MicroRNA Expression in the Aqueous Humor of Patients with Diabetic Macular Edema. Int. J. Mol. Sci..

[12] Cho H., Hwang M., Hong E.H., Yu H., Park H.H., Koh S.H., Shin Y.U. (2020). Micro-RNAs in the aqueous humour of patients with diabetic macular oedema. Clin. Exp. Ophthalmol..

[13] Castro-Villegas C., Pérez-Sánchez C., Escudero A., Filipescu I., Verdu M., Ruiz-Limón P., Aguirre M.A., Jiménez-Gomez Y., Font P., Rodriguez-Ariza A. (2015). Circulating miRNAs as potential biomarkers of therapy effectiveness in rheumatoid arthritis patients treated with anti-TNFα. Arthritis Res. Ther..

[14] Citron F., Segatto I., Musco L., Pellarin I., Rampioni Vinciguerra G.L., Franchin G., Fanetti G., Miccichè F., Giacomarra V., Lupato V. (2021). miR-9 modulates and predicts the response to radiotherapy and EGFR inhibition in HNSCC. EMBO Mol. Med..

[15] Chen X., Yao Y., Yuan F., Xie B. (2020). Overexpression of miR-181a-5p inhibits retinal neovascularization through endocan and the ERK1/2 signaling pathway. J. Cell Physiol..

[16] Dentelli P., Rosso A., Orso F., Olgasi C., Taverna D., Brizzi M.F. (2010). microRNA-222 controls neovascularization by regulating signal transducer and activator of transcription 5A expression. Arter. Thromb. Vasc. Biol..

[17] Robinson P.M., Chuang T.D., Sriram S., Pi L., Luo X.P., Petersen B.E., Schultz G.S. (2013). MicroRNA signature in wound healing following excimer laser ablation: Role of miR-133b on TGFβ1, CTGF, SMA, and COL1A1 expression levels in rabbit corneal fibroblasts. Investig. Ophthalmol. Vis. Sci..

[18] Babae N., Bourajjaj M., Liu Y., Van Beijnum J.R., Cerisoli F., Scaria P.V., Verheul M., Van Berkel M.P., Pieters E.H., Van Haastert R.J. (2014). Systemic miRNA-7 delivery inhibits tumor angiogenesis and growth in murine xenograft glioblastoma. Oncotarget.

[19] Vader M.J.C., Schauwvlieghe A.M.E., Verbraak F.D., Dijkman G., Hooymans J.M.M., Los L.I., Zwinderman A.H., Peto T., Hoyng C.B., van Leeuwen R. (2020). Comparing the Efficacy of Bevacizumab and Ranibizumab in Patients with Diabetic Macular Edema (BRDME): The BRDME Study, a Randomized Trial. Ophthalmol. Retin..

[20] Giani A., Cigada M., Choudhry N., Deiro A.P., Oldani M., Pellegrini M., Invernizzi A., Duca P., Miller J.W., Staurenghi G. (2010). Reproducibility of retinal thickness measurements on normal and pathologic eyes by different optical coherence tomography instruments. Am. J. Ophthalmol..

[21] Ruijter J.M., Ramakers C., Hoogaars W.M., Karlen Y., Bakker O., van den Hoff M.J., Moorman A.F. (2009). Amplification efficiency: Linking baseline and bias in the analysis of quantitative PCR data. Nucleic Acids Res..

[22] Klaassen I., Hughes J.M., Vogels I.M., Schalkwijk C.G., Van Noorden C.J., Schlingemann R.O. (2009). Altered expression of genes related to blood-retina barrier disruption in streptozotocin-induced diabetes. Exp. Eye Res..

[23] del Toro R., Prahst C., Mathivet T., Siegfried G., Kaminker J.S., Larrivee B., Breant C., Duarte A., Takakura N., Fukamizu A. (2010). Identification and functional analysis of endothelial tip cell-enriched genes. Blood.

[24] Rocha S.F., Schiller M., Jing D., Li H., Butz S., Vestweber D., Biljes D., Drexler H.C., Nieminen-Kelhä M., Vajkoczy P. (2014). Esm1 modulates endothelial tip cell behavior and vascular permeability by enhancing VEGF bioavailability. Circ. Res..

[25] Yang C., Tahiri H., Cai C., Gu M., Gagnon C., Hardy P. (2018). microRNA-181a inhibits ocular neovascularization by interfering with vascular endothelial growth factor expression. Cardiovasc. Ther..

[26] Kamal N.N.M., Shahidan W.N.S. (2019). Non-Exosomal and Exosomal Circulatory MicroRNAs: Which Are More Valid as Biomarkers?. Front. Pharmacol..

